# Psychometric Properties and Validation of the Arabic Social Media Addiction Scale

**DOI:** 10.1155/2015/291743

**Published:** 2015-08-11

**Authors:** Jamal Al-Menayes

**Affiliations:** Department of Mass Communication, College of Arts, Kuwait University, P.O. Box 23558, 13096 Safat, Kuwait

## Abstract

This study investigated the psychometric properties of the Arabic version of the SMAS. SMAS is a variant of IAT customized to measure addiction to social media instead of the Internet as a whole. Using a self-report instrument on a cross-sectional sample of undergraduate students, the results revealed the following. First, the exploratory factor analysis showed that a three-factor model fits the data well. Second, concurrent validity analysis showed the SMAS to be a valid measure of social media addiction. However, further studies and data should verify the hypothesized model. Finally, this study showed that the Arabic version of the SMAS is a valid and reliable instrument for use in measuring social media addiction in the Arab world.

## 1. Introduction

The Diagnostic and Statistical Manual of Mental Disorder (DSM-5) used by many mental health practitioners does not define social media addiction as a disorder. However, in the fifth edition of DSM-5, Internet Gaming Disorder is identified in Section 3 as a condition warranting more clinical research and experience before it might be considered for inclusion in the main book as a formal disorder. Regardless, a vast number of people show what are apparently symptoms of addiction to Cyberspace. This phenomenon seems to afflict young individuals, in particular, with evidence revealing students whose academic achievement is undermined as they spend increasing amount of time online [1, 2]. Some also experience adverse health consequences resulting from lack of sleep due to being online for a long time, particularly late at night [3].

Research into Internet addiction has grown significantly since the mid-1990s, especially as more and more cases among college students have been detected by university healthcare staff [4]. The terminology, to describe this occurrence, varies greatly in the literature. In addition to “Internet addiction,” concepts such as “Internet dependency,” “compulsive Internet use,” “problematic Internet use,” “dysfunctional Internet use,” and “pathological Internet use” have been used to describe what is fundamentally the same phenomenon [5]. For the current paper, I will use “Internet addiction” chiefly due to its wide adoption in the research in this area.

International research has produced varying results of the prevalence of Internet addiction. A study in the UK, for example, found Internet addiction to be widespread among 18% of young people [6]. A study in Italy found the rate to be only 0.8% [7]. Furthermore, a large sample survey in China puts the rate at 12% among male and 5% among female college students [8]. Internet addiction is not just limited to college students; it also extends to high school as well as middle school students. A longitudinal survey conducted in Hong Kong found the incidence rate of Internet addiction as high as 26.7% among high school students [9].

As for the amount of time spent online, research indicates that individuals, who consider themselves Internet addicts, reported that it varies greatly from 8.5 hours per week to 21.2 hours per week [10]. Other studies found a positive correlation between the amount of time spent online and the severity of the symptoms of Internet addiction [11, 12].

Regarding users' psychological profile, studies have revealed a correlation between depression, locus of control, loneliness, social anxiety, and self-esteem and Internet addiction [13, 14]. Whang et al., for example, found that Internet addicts had a higher degree of loneliness and depression compared to nonaddicts [15]. Other findings showed that computer self-efficacy was a significant correlate of problematic Internet use. Internet addiction was also related to poor mental health and low self-esteem in adolescents [16].

## 2. Social Media Addiction

Online addiction is virtually indistinguishable from social media addiction with the single difference of the latter being used primarily on mobile devices. While none of the previous researches dealt with the use of mobile social media as such, it is safe to say that the results of computer related Internet addiction also apply to mobile Internet since they both use the same medium essentially. The introduction of anytime, anywhere Wi-Fi in mobile phones and the prevalence of free social media apps make it impossible to differentiate them from personal computers when it came to Internet addiction. Furthermore, as their name signifies, mobile phones are portable providing easy access to the Internet regardless of place and time. Portability makes them the ideal medium for Internet addicts.

Mobile social media offer a large number of experiences from a psychological perspective, each with potent features that can lead to problem behavior. For example, the extrovert might spend much time on Facebook, repeatedly going over their profile to see the number of “likes” their latest post received. For others, with a narcissistic predisposition, Instagram may prove to be an addictive medium for them to display themselves to others with “selfies.” Social anxiety can also lead to social media addiction. The fear of missing out (FOMO) can be the key reason for repeated social media use irrespective of time of day to the detriment of other activities [17].

“Mobile phone addiction” is sometimes used to distinguish it from the concept of Internet addiction [18, 19]. Most of the traditional studies of online addiction do not address problematic mobile phone use. Mobile phones today offer access to almost all Internet applications along with voice and video calls, text messaging, and video recording. Also, there is a myriad of engaging apps designed especially for small screens, but their results can also be shown on any screen. Furthermore, they have the added dimension of being always available, unlike a desktop or even a laptop.

The mobile phone can be used while walking and riding on public transportation and even while driving. These “micro time slots” in which people can engage in a multitude of online activities were not previously available. Micro time slots can lead to obsessive mobile phone usage and can interfere with face-to-face interaction and harm academic performance [20].

Studies on problematic mobile media usage are limited but have attracted increasing attention recently. A study of Taiwanese female university students, for example, found that students, who scored high on a test of mobile phone addiction, showed more extraversion and anxiety and somewhat lower self-esteem [21]. Women seem to be more susceptible to mobile phone addiction than men. Another study found that problematic cell phone usage is associated with age, depression, extraversion, and low impulse control [22]. Cellphone addiction also varied considerably across male and female users with women spending far more time using their cell phones and showing more signs of addiction than men [23].

Another feature of mobile phones that may prove to be of particular significance to addictive behavior is “texting” either directly or through social media such as Twitter and similar applications. Recent surveys indicate that young people are starting to discard Facebook in favor of Twitter, particularly as their parents create accounts and ask to be “friended” [24]. These types of applications are growing and enabling more and more features such as Vine, which allows users to create six-second videos to share with followers. The common feature of these applications is their “stickiness,” the tendency to have users utilize the app frequently. Stickiness is a result of their business models that rely on the growing mass of data on user behavior to share with advertisers for targeted marketing.

## 3. The Psychometrics of Social Media Addiction Measurement

The scale used the most in Internet addiction research is Young's Internet Addiction Test (IAT) [25]. Several investigations have examined the psychometric properties of the IAT. Widyanto and McMurran [26] performed a factor analysis on the IAT. Their relatively small sample of 86 consisted of 29 males and 57 females. The subjects age ranged from 13 to 67 years. The researchers extracted six factors from the IAT: salience (five items), excessive use (five items), neglect of work (three items), anticipation (two items), self-control (five items), and neglect of social life (two items). These factors accounted for 35.80 percent, 9.02 percent, 6.51 percent, 6.02 percent, 5.55 percent, and 5.21 percent of the variance, respectively. Despite the small sample size, Widyanto and McMurran suggested that the IAT has the potential to be a good foundation for constructing a valid measurement. Chang and Law [27] performed factor analysis of IAT using a sample of 410 students from eight universities in Hong Kong. The results led the researchers to eliminate three items due to poor loadings. The researchers then identified three factors: withdrawal and social problems (9 items), time management and performance (5 items), and reality substitution (3 items). These factors accounted for 24.29 percent, 20.80 percent, and 10.64 percent of the variance, respectively.

Jelenchick et al. conducted a psychometric analysis of the IAT with results from a sample of 215 US undergraduates from two universities. They uncovered two factors, “dependent” use and “excessive use,” accounting for 73 percent and 17 percent of the variance, respectively. Consequently, they concluded that the IAT is a valid measurement for assessing Internet addiction in US college students [28]. Khazaal et al. studied the psychometric properties of a French version of the IAT with a sample of 246 adults composed of undergraduate medical students and volunteers from the community [29]. The participants ages ranged from 18 to 54 years (81 males and 165 females). They reported one factor that had good loadings and goodness of fit. Finally, Hawi attempted to validate an Arabic version of the IAT from a sample of 817 from intermediate and secondary school students aged 10–22 years. His analysis yielded one factor that fits the data well, something similar to the French version [30].

## 4. Research Questions

The present study sought to examine the psychometric properties of an Arabic version of the IAT customized to assess social media, in particular, not the entire Internet space. The word Internet was replaced by “social media” to arrive at Social Media Addiction Scale (SMAS). More specifically, the research aims to answer these questions:

1. What is the internal consistency of the SMAS?
2. How many factors underlie the SMAS, and what are they?
3. To what degree is the SMAS concurrently valid?

## 5. Method

### 5.1. Participants

A total of 1327 undergraduate students enrolled in communication courses in a large state university completed a self-administered survey questionnaire over a period of four months during the academic year in 2014. The sample was selected based on convenience and therefore it is not a probability sample. Students were assured of anonymity and confidentiality, and participation was voluntary. A research assistant distributed the questionnaires at the beginning of each class and the time it took to fill it out is just under ten minutes. The age of the respondents ranged from 18 to 31 with 96% ranging from 18 to 25 years of age. The mean age of the participants in the study was 21.87 years. The participants were 395 (29.8%) males and 931 (70.2%) females. This gender distribution reflects the enrollment profile of the university student body that is 70% female. The self-administered questionnaires were distributed during regularly scheduled class sessions.

### 5.2. Instrument

The SMAS consisted of 14 items adapted from the IAT to fit the context of social media usage. The items were rated on a five-point Likert scale: strongly agree, agree, neutral, disagree, and strongly disagree, scored 5, 4, 3, 2, and 1, respectively. The sample size is sufficient for a scale consisting of 14 items.

### 5.3. Social Media Engagement Questionnaire (SMEQ)

The SMEQ provides a way of quantifying levels of personal use, measuring the extent to which people's key daily activities tend to involve social media. It consists of five items measuring social media usage patterns throughout different parts of the day from the time one gets up in the morning to the time they go to bed [31]. The items were rated on a seven-point scale ranging from 0 (never) to 7 (seven times) (Table 2).

## 6. Results

Table 1 shows the wording of all fourteen items in addition to the means and standard deviations. Next, the 14 items of the SMAS were subjected to exploratory factor analysis using principal access factoring (PAF) extraction method (Table 3). The analysis produced three factors. The first included five items explaining 31.06 percent of the variance, an Eigenvalue of 4.34, and Cronbach's alpha of 0.75. I call this factor* Social Consequences of SM* since the items seem to reflect how SM usage affects one's daily life activities. The second factor contained three items explaining 11.82 percent of the variance, an Eigenvalue of 1.65, and Cronbach's alpha of 0.66. I call this factor* Time Displacement* since the items reflect the time dimension as it relates to SM usage. The third factor had four items explaining 8.84 percent of the variance, Eigenvalue of 1.23, and Cronbach's alpha of 0.61. I call it* Compulsive Tendencies* since the items seem to reflect that dimension.

All labels are tentative pending further investigation of the subject based on thorough theoretical grounding. The three-factor result is inconsistent with the Arabic version of the IAT that had a single factor even though it contained far more items [30]. The result is also different from findings in the US (two factors), the UK (six factors), and France (one factor) [31, 32] Discrepancies are most likely due to sampling procedures and differences inherent in the study sample for each location. These factors can be demographic characteristics, cultural norms, or socioeconomic factors.

To assess the concurrent validity of the SMAS a correlation matrix was needed to assess the relationship between it and a criterion variable that has a theoretical link to it. To do this, a correlation was calculated between the three factors of SMAS and Social Media Engagement Questionnaire (SMEQ). The SMEQ measures the extent to which people's key daily activities tend to involve social media. The SMEQ consists of five items that were all included in the same survey [33].  Table 4 shows the wording of the items along with means and standard deviations. We would expect the SMEQ to be positively related to SMAS since they both purport to measure the extent of usage. The three-factor scores of the SMAS were correlated with the five items of the SMEQ.  Table 4 shows results of this analysis. As can be seen, out of a total of 15 combinations all items were positively correlated save one. That would be a 93 percent success rate which is very good. This leads to the conclusion that the SMAS is a valid measure of addiction in our sample.

## 7. Conclusion

This study examined the psychometric properties of the Arabic version of the SMAS. SMAS is a variant of IAT customized to measure addiction to social media instead of the Internet as a whole. First, the exploratory factor analysis showed that a three-factor model fits the data well. Second, concurrent validity analysis showed the SMAS to be a valid measure of social media addiction. However, further research and data should verify the hypothesized model. Finally, this study showed that the Arabic version of the SMAS is a valid and reliable instrument for use in measuring social media addiction in the Arab world.

## Figures and Tables

**Table 1:** Means and standard deviations of Social Media Addiction Scale (original in Arabic).

Variable	M	SD
(1) I often find myself using social media longer than intended.	4.20	.95
(2) I often find life to be boring without social media.	3.98	1.09
(3) I often neglect my schoolwork because of my usage of social media.	3.25	1.22
(4) I get irritated when someone interrupts me when I'm using social media.	3.25	1.20
(5) Several days could pass without me feeling the need to use social media.	2.66	1.32
(6) Time passes by without me feeling it when I am using social media.	4.18	.94
(7) I find it difficult to sleep shortly after using social media.	2.89	1.28
(8) I will be upset if I had to cut down the amount of time I spend using social media.	2.89	1.19
(9) My family complain frequently of my preoccupation with social media.	2.90	1.37
(10) My school grades have deteriorated because of my social media usage.	2.44	1.24
(11) I often use social media while driving.	2.66	1.36
(12) I often cancel meeting my friends because of my occupation with social media.	1.67	.97
(13) I find myself thinking about what happened in social media when I am away from them.	3.03	1.24
(14) I feel my social media usage has increased significantly since I began using them.	3.67	1.15

*n* = 1327; range = 1–5.

**Table 2:** Means and standard deviations of Social Media Engagement Questionnaire (SMEQ) (original in Arabic).

Variable	M	SD
*In the past week *		
(1) How many times have you used social media 15 minutes before going to bed?	4.94	2.35
(2) How many times have you used social media during breakfast in the morning?	3.07	2.64
(3) How many times you used social media while eating dinner at night?	3.67	2.62
(4) How many times you used social media within 15 minutes of waking up in the morning?	4.26	2.64
(5) How many times you used social media while eating lunch?	3.00	2.64

*n* = 1327; range = 1–7.

**Table 3:** Exploratory factor analysis of Social Media Addiction Scale (SMAS).

Factors	Mean	SD	1	2	3
*Factor 1: Social Consequences *					
(1) I get irritated when interrupted using SM.	2.90	1.20	0.513		
(2) My family complain because of SM.	2.44	1.37	0.561		
(3) School grades deteriorated because of SM.	1.67	1.24	0.669		
(4) I often cancel meetings because of SM.	3.03	0.97	0.623		
(5) I find myself thinking about SM.		1.24	0.531		

*Factor 2: Time Displacement *					
(6) I find myself using SM longer than intended.	4.20	0.95		0.637	
(7) Time passes without feeling using SM.	4.18	0.94		0.660	
(8) I neglect my schoolwork because of SM.	3.25	1.22		0.550	

*Factor 3: Compulsive Feelings *					
(9) I find life boring without SM.	3.98	1.09			0.616
(10) Days pass by without the need to use SM.^*∗*^	2.66	1.32			−0.525
(11) I will be upset if I cut down SM use.	2.89	1.19			0.522
(12) My SM use increased since when I started.	3.67	1.15			0.550

Eigenvalue			4.34	1.65	1.23
% of the variance explained			31.06	11.82	8.84
Cronbach's alpha			0.75	0.66	0.61

Notes: loadings < 0.50 were suppressed. ^*∗*^Item wording was reversed for reliability analysis.

**Table 4:** Concurrent validity by correlating social media addiction factors with Social Media Engagement Questionnaire (SMEQ) scale.

Variable	SMEQ1	SMEQ2	SMEQ3	SMEQ4	SMEQ5
Addiction factor 1	.04	.20^*∗∗*^	.25^*∗∗*^	.12^*∗∗*^	.24^*∗∗*^
Addiction factor 2	.25^*∗∗*^	.25^*∗∗*^	.30^*∗∗*^	.28^*∗∗*^	.23^*∗∗*^
Addiction factor 3	.31^*∗∗*^	.32^*∗∗*^	.35^*∗∗*^	.31^*∗∗*^	.32^*∗∗*^

Addiction factors are saved regression scores from factor analysis. Entries are zero-order correlation coefficients, ^*∗∗*^ *p* < .01.
